# Bone Metastases Lesion Segmentation on Breast Cancer Bone Scan Images with Negative Sample Training

**DOI:** 10.3390/diagnostics13193042

**Published:** 2023-09-25

**Authors:** Yi-You Chen, Po-Nien Yu, Yung-Chi Lai, Te-Chun Hsieh, Da-Chuan Cheng

**Affiliations:** 1 Department of Biomedical Imaging and Radiological Science, China Medical University, Taichung 404, Taiwan; u111202701@cmu.edu.tw (Y.-Y.C.); u111202703@cmu.edu.tw (P.-N.Y.); 2 Department of Nuclear Medicine, Feng Yuan Hospital, Ministry of Health and Welfare, Taichung 420, Taiwan; daniellai999@hotmail.com; 3 Department of Nuclear Medicine and PET Center, China Medical University Hospital, Taichung 404, Taiwan

**Keywords:** bone metastasis segmentation, Double U-Net, pre-train, negative mining, transfer learning, deep learning

## Abstract

The use of deep learning methods for the automatic detection and quantification of bone metastases in bone scan images holds significant clinical value. A fast and accurate automated system for segmenting bone metastatic lesions can assist clinical physicians in diagnosis. In this study, a small internal dataset comprising 100 breast cancer patients (90 cases of bone metastasis and 10 cases of non-metastasis) and 100 prostate cancer patients (50 cases of bone metastasis and 50 cases of non-metastasis) was used for model training. Initially, all image labels were binary. We used the Otsu thresholding method or negative mining to generate a non-metastasis mask, thereby transforming the image labels into three classes. We adopted the Double U-Net as the baseline model and made modifications to its output activation function. We changed the activation function to SoftMax to accommodate multi-class segmentation. Several methods were used to enhance model performance, including background pre-processing to remove background information, adding negative samples to improve model precision, and using transfer learning to leverage shared features between two datasets, which enhances the model’s performance. The performance was investigated via 10-fold cross-validation and computed on a pixel-level scale. The best model we achieved had a precision of 69.96%, a sensitivity of 63.55%, and an F1-score of 66.60%. Compared to the baseline model, this represents an 8.40% improvement in precision, a 0.56% improvement in sensitivity, and a 4.33% improvement in the F1-score. The developed system has the potential to provide pre-diagnostic reports for physicians in final decisions and the calculation of the bone scan index (BSI) with the combination with bone skeleton segmentation.

## 1. Introduction

According to the gender statistics database published by the Gender Equality Committee of the Executive Yuan in Taiwan in 2023, breast cancer was ranked first among the top 10 cancer incidence rates in 2020 [1]. Breast cancer, prostate cancer, lung cancer, and other prevalent cancers account for more than 80% of cases of metastatic bone disease. For patients with breast cancer, late-stage bone metastasis is prone to occur, significantly reducing the prognosis of the patients. A study by Coleman and Rubens reported bone metastasis in 69% of breast cancer patients who died between 1979 and 1984, out of a total of 587 patients [2]. Bone metastasis in breast cancer most commonly occurs in the spine, followed by the ribs and sternum [3]. Radiologically, bone metastases in breast cancer are predominantly osteolytic, leading to severe complications such as bone pain, pathological fractures, spinal cord compression, hypercalcemia, and bone marrow suppression. Therefore, the early detection and treatment of bone metastasis in breast cancer patients are crucially important.

Current methods for detecting breast cancer metastasis include the clinical observation of distant organ involvement, organ biopsies, diagnostic imaging, and serum tumor markers. One of the primary imaging techniques used in clinics for bone metastasis diagnosis is the whole-body bone scan (WBBS) with vein injection using the Tc-99m MDP tracer [4,5]. WBBS offers the advantages of whole-body examination, cost-effectiveness, and high sensitivity, making it a preferred modality for bone metastasis screening [6]. Unlike X-radiography (XR) and computed tomography (CT) images, which can only detect changes in bone when there is approximately 40–50% mineralization [7], bone scans exhibit higher sensitivity in detecting bone changes, capable of detecting alterations as low as 5% in osteoblast activity. The reported sensitivity and specificity of skeletal scintigraphy for bone metastasis detection are 78% and 48%, respectively [8].

The bone scan index (BSI) is an image biomarker utilized in WBBS to evaluate the severity of bone metastasis in cancer patients. It enables a quantification on the degree of tumor involvement in the skeleton [9,10]. BSI is used for observing disease progression or treatment response. The commercial software EXINI bone (version 1 and version 2, including subversions), developed by EXINI Diagnostics AB, incorporates aBSI (automated bone scan index) technology for the comprehensive automated quantitative assessment of bone scan images [11]. In [11], there exists a strong correlation between manual and automated BSI assessment values (ρ = 0.80), which further strengthens (ρ = 0.93) when cases with BSI scores exceeding 10 (1.8%) are excluded. This indicates that automated BSI calculations can deliver clinical value comparable to manual calculations. Shimizu et al. has proposed an image interpretation system based on deep learning [12], using BtrflyNets for the hotspot detection of bone metastasis and bone segmentation, followed by automatic BSI calculation. The aBSI technology has now become a clinically valuable tool. Nevertheless, there are still challenges regarding recognition performance (sensitivity and precision) in this technique.

Cheng et al. applied a deep convolutional neural network (D-CNN) for the object detection of bone metastasis from prostate cancer in bone scan images [13]. Their investigation specifically focused on the chest and pelvic regions, and the sensitivity and precision for detecting and classifying chest bone metastasis were determined using bounding boxes to be 0.82 ± 0.08 and 0.70 ± 0.11, respectively. Regarding pelvic bone metastasis classification, the reported sensitivity and specificity were 0.87 ± 0.12 and 0.81 ± 0.11, respectively. Cheng et al. conducted a more detailed study on chest bone metastasis in prostate cancer patients [14]. The average sensitivity and precision for detecting and classifying chest bone metastasis based on lesion locations are reported as 0.72 ± 0.04 and 0.90 ± 0.04, respectively. For classifying chest bone metastasis based on patient-level outcomes, the average sensitivity and specificity are found to be 0.94 ± 0.09 and 0.92 ± 0.09, respectively. Patents filed by Cheng et al. are referenced as [15], which leverage deep learning for the identification of bone metastasis in prostate cancer bone scan images. Since they use bounding boxes, they are unable to calculate BSI.

In a related study [16], a neural network (NN) model based on U-Net++ is proposed for the automated segmentation of metastatic lesions in bone scan images. The anterior–posterior and posterior–anterior views are superimposed, and image segmentation is exclusively performed on the chest region of whole-body bone scan images. The achieved average F1-score is 65.56%.

In this study, we modified the Double U-Net [17] as the fundamental architecture to perform bone metastases segmentation on WBBS. We explored various methods to enhance network performance, including background pre-processing, adding negative samples, and transfer learning. We used Otsu thresholding [18] and negative mining [14] methods for background pre-processing and generating negative samples. Background pre-processing helped eliminate unnecessary background information, while adding negative samples reduced the model’s false positive rate. Both of these methods did not require manual labeling or modification, saving time and manpower. Previous studies in the same field [16,19,20,21,22,23] focused only on segmenting bone metastases in specific regions (chest or pelvis) and could only predict either the anterior or posterior view. The datasets we used only excluded the non-metastatic-prone areas below the knees and could simultaneously segment images in both the anterior and posterior views. In comparison, our model was able to provide a more comprehensive assessment of bone metastasis images.

The following points summarize the contributions of this paper:

- We discuss the challenges of lesion segmentation in breast cancer bone scan images.
- We compare and discuss the state-of-the-art methods in the same research field.
- Our experiments have shown that background pre-processing significantly improves a model’s performance and adding negative samples enhances model precision. Both methods do not require manual labeling or label modification, saving time and manpower.
- Our segmentation model offers greater comprehensiveness. It can perform lesion segmentation on WBBS images, predicting both anterior and posterior views simultaneously.

## 2. Related Work

Deep learning has found numerous applications in cancer detection tasks. The authors of Ref. [24] proposed an improved SIFT descriptor with Harris corner to form Bag-Of-Words features in image representation. This study made a significant contribution to medical image classification tasks. For skin lesions, the authors of Ref. [25] conducted a comprehensive comparative study of U-Net and attention-based methods for dermatological image segmentation, aiding in the diagnosis of skin lesions. The authors of Ref. [26] introduced an enhanced deep learning model, SBXception, based on the Xception network to improve skin cancer classification. In the realm of MRI, the authors of Ref. [27] presented a weighted ensemble deep learning model for brain tumor classification. The authors of Ref. [28] explored five machine learning techniques to deepen the understanding of brain tumor classification and enhance its scope and significance.

Some early work has been carried out on automatic segmentation of metastatic lesions using bone scan images [29,30,31,32,33]. The trend of using deep learning for bone scintigraphy image analysis is becoming increasingly evident. In classification tasks, the authors of Ref. [34] introduced an improved ResNet model that combines convolutional block attention module and contextual transformer attention mechanisms to achieve the accurate classification of SPECT images [35] based their work on widely used deep networks, including VGG, ResNet, and DenseNet, by fine-tuning their parameters and structures or by customizing new network architectures. The proposed classifiers performed well in identifying bone metastases through SPECT imaging. The authors of Ref. [36] presented an automated bone metastasis diagnostic model based on multi-view images. The authors of Ref. [37] introduced a new framework in this work, which included data preparation and image classification, for automatically classifying scintigraphy images collected from patients clinically diagnosed with lung cancer.

In object detection tasks, the authors of Ref. [38] employed scaled-YOLOv4 and Detectron2 object detection networks for bone metastasis localization in breast cancer patient nuclear imaging data and for detecting degenerative and pathological findings in whole-body scintigraphy scans. The authors of Ref. [39] proposed an automatic lesion detection model based on single shot multibox object detector for the automatic detection of lung cancer bone metastases in low-resolution SPECT bone scintigraphy images. The authors of Ref. [14] applied D-CNN for object detection of prostate cancer bone metastases in the chest and pelvic regions. As object detection uses bounding boxes, it cannot calculate the BSI as a subsequent quantitative measure.

Compared to classification and object detection tasks, segmentation tasks are more challenging. The authors of Ref. [19] introduced a model called MaligNet, which semantically segments abnormal hotspots in a semi-supervised manner and classifies bone cancer metastases in the chest region. The authors of Ref. [20] built a segmentation model based on U-Net and Mask R-CNN networks by fine-tuning their architectures for identifying and segmenting metastatic hotspots in bone SEPCT images. The authors of Ref. [21] added a methods attention mechanism on top of the original U-Net network’s skip connections to enhance feature selection, allowing for the automatic identification and segmentation of bone metastases. The authors of Ref. [16] proposed a neural network model based on U-Net++ for the automatic segmentation of metastatic lesions in bone scan images. The authors of Ref. [22] introduced an improved UNet3+ network that combines attention mechanisms for the automatic segmentation of bone metastatic lesions. The authors of Ref. [23] presented a bone imaging focus segmentation algorithm based on the Swin Transformer, which uses the swin transformer as the backbone network for extracting feature information from bone images. In current research in the same field, segmentation tasks are limited to predicting specific local regions, such as the chest or pelvis, and they cannot simultaneously predict both anterior and posterior views.

## 3. Materials and Methods

### 3.1. Materials

**Figure 1:**
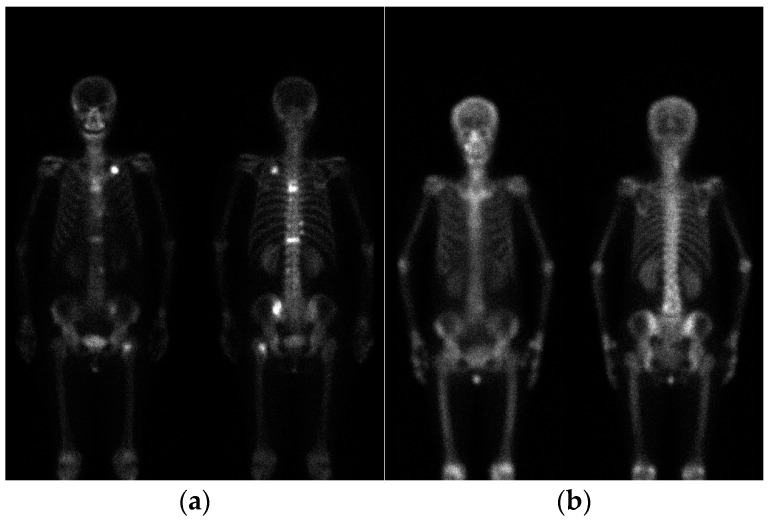
Bone scan images of breast cancer patients. (**a**) With metastasis; (**b**) without metastasis.

In this study, we collected 200 bone scan images from the Department of Nuclear Medicine of China Medical University Hospital. The details of the bone scan images are provided in Table 1. Specifically, D1 is defined as 90 images from breast cancer patients with bone metastasis. D2 is defined as 10 images from breast cancer patients without bone metastasis. D3 is defined as 50 images from prostate cancer patients with bone metastasis. D4 is defined as 50 images from prostate cancer patients without bone metastasis. Figure 1 shows bone scan images of breast cancer patients. This study has been approved by the Institutional Review Board (IRB) of China Medical University and Hospital Research Ethics Committee (CMUH106-REC2-130), approved on 27 September 2017.

The WBBS process can be described as follows. Patients undergo WBBS with a gamma camera (Millennium MG, Infinia Hawkeye 4, or Discovery NM/CT 670 system; GE Healthcare, Waukesha, WI, USA). Bone scans are acquired 2–4 h after the intravenous injection of 740–925 MBq (20–25 mCi) of technetium-99m methylene diphosphonate (Tc-99m MDP) with an acquisition time of 10–15 cm/min. The collected WBBS images are saved in DICOM format. The raw images include anterior–posterior (AP) and posterior–anterior (PA) views, with a matrix size of 1024 × 256 pixels.

### 3.2. Image Labeling

**Figure 2:**
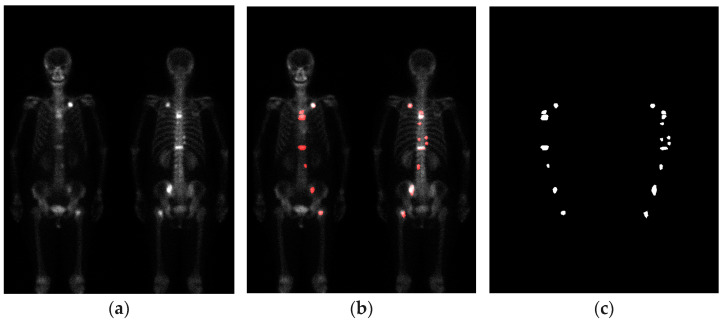
The schematic of the manually annotated results. (**a**) Bone scan image; (**b**) overlay of bone scan image with ground truth; (**c**) ground truth.

To facilitate labeling the bone scan images, the Labelme (version 4.5.9) software is used as the annotation tool. The manual annotation of bone metastasis images is carried out under the guidance and supervision of nuclear medicine physicians. This process is very time-consuming. The outputs generated by the Labelme software are saved in JSON format, and then converted to the PNG format. Figure 2 represents a schematic of the manually annotated results.

### 3.3. Image Pre-Processing

**Figure 3:**
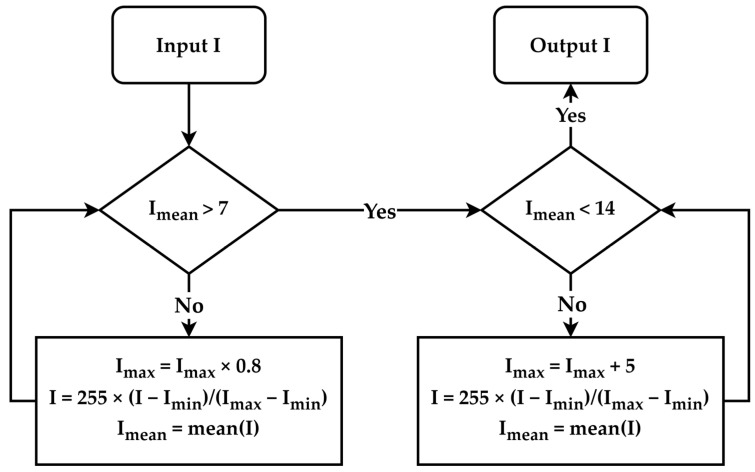
Flowchart of brightness normalization.

The raw images possess a large memory size and the DICOM format is not directly suitable for neural network training. Moreover, the raw images exhibit variations in brightness and contrast levels. Thus, the pre-processing of the raw images becomes imperative. The detection of the body range was accomplished using the projection profile, followed by the extraction of two views with dimensions of 950 × 256 pixels through cutting and centering. No scaling or other transformations were applied during this process. We utilized the brightness normalization method proposed in [14] for brightness pre-processing. This method uses a linear transformation to adjust the dynamic range of an image, with the objective of controlling the average intensity of each image within the range of (7, 14). The algorithm for the linear transformation is illustrated in Figure 3. The region below the knees, which is uncommon for bone metastasis, was excluded from the calculation of BSI. To obtain the region above the knees, pixels beyond row 640 were eliminated, resulting in two views with a spatial resolution of 640 × 256 pixels each. Finally, the pre-processed AP (anterior–posterior) and PA (posterior–anterior) view images were horizontally merged, generating images with a spatial resolution of 640 × 512 pixels.

### 3.4. Positive and Negative Samples

According to previous research [14], adding negative samples to the training dataset helps reduce false positives and improve model precision. In this study, we also used negative samples to enhance the model’s performance. Positive samples are defined as images with bone metastases (D1 and D3), while negative samples are defined as images without bone metastases (D2 and D4).

For the bone metastasis segmentation task in WBBS images, the background significantly interferes with network training. In this scenario, the background not only includes air but also the non-metastatic (NM) human body regions. Intuitively, NM regions of the human body contain information but do not contain air. Therefore, an alternative approach is to filter out the air to extract NM human body regions to generate NM masks. The generation of NM masks involves two methods, which we briefly explain below.

The Otsu thresholding method is used to generate NM masks for both positive and negative samples. It is important to note that the metastatic (M) regions must be manually excluded from positive samples beforehand. Otsu thresholding can automatically determine the threshold that separates air from the human body.

The negative mining method for generating NM masks involves two steps. First, the baseline network is trained using only positive samples. After training, this model is used to predict negative samples. Since negative samples do not have bone metastatic lesions, all segmentation results produced are false positives. These false positive segmentation results are then treated as NM regions to generate NM masks. The same model is also used to predict positive samples. It is worth noting that the metastatic (M) regions in the positive samples must be manually excluded beforehand.

The initial classes include background and metastasis. After generating NM masks using the two methods mentioned above, the number of classes increases from the original two to three, now including air-background (BG), non-metastatic (NM), and metastatic (M) classes.

### 3.5. Transfer Learning

Transfer learning is a widely used technique in neural networks to increase their performance. Before applying transfer learning, two crucial factors need to be considered: (1) the size of the target dataset and (2) the similarity between the target dataset and the pre-training dataset.

In this study, the Double U-Net network model was pre-trained using the D3 and D4 datasets. We chose a pre-training dataset that contains highly similar bone scan images of prostate cancer. Subsequently, the model was fine-tuned using the target dataset consisting of breast cancer bone scan images. By leveraging transfer learning and selecting a pre-training dataset closely related to the target dataset, our goal was to utilize shared features between the two datasets to enhance the model’s performance on the current specific task.

### 3.6. Neural Network Model

**Figure 4:**
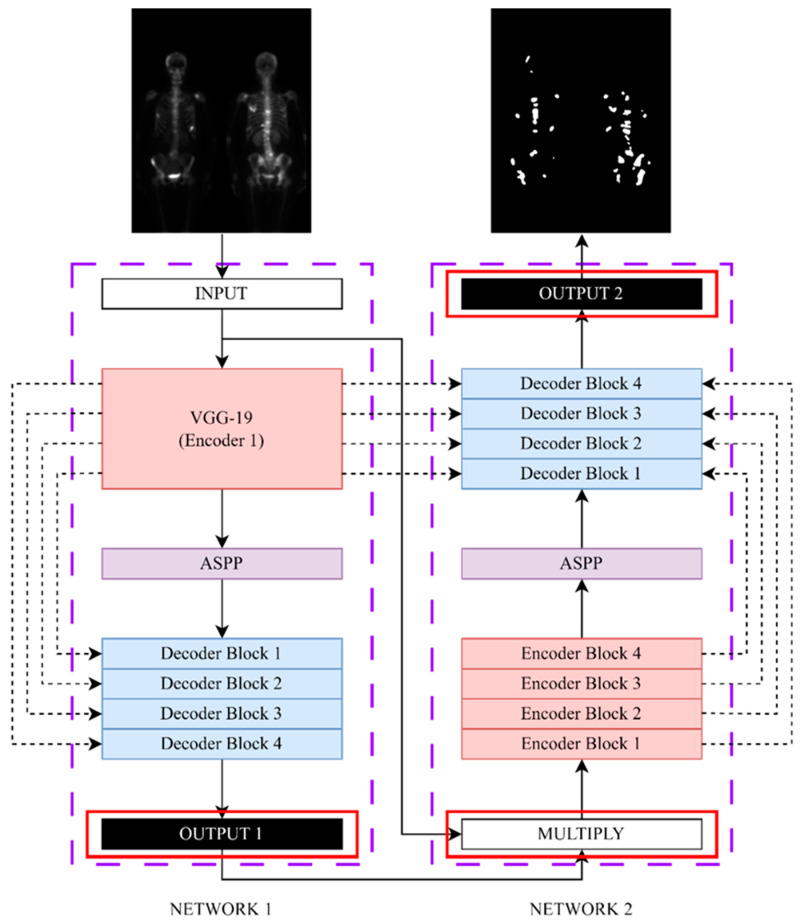
The modified architecture diagram of Double U-Net, the baseline network.

We adopted the Double U-Net architecture as our network framework. The original Double U-Net architecture was developed for binary segmentation tasks, which we refer to as the baseline network. To adapt the Double U-Net architecture for multi-class segmentation, we modified its network structure following the method described in our previous research [40]. Figure 4 illustrates the modified network architecture. We changed the output layer of network 1 to obtain a SoftMax activation function, enabling it to perform multi-class segmentation. With this modification, the Double U-Net architecture can handle three-class segmentation tasks involving the BG, NM, and M regions.

### 3.7. Loss Function

The selection of an appropriate loss function is a critical aspect in the design of deep learning architectures for image segmentation tasks, as it greatly impacts the learning dynamics of the algorithm. In our study, we consider two loss functions: the Dice loss (Equation (1)), as originally proposed in [17], and the Focal Tversky loss (Equation (2)). By comparing these loss functions, we aim to explore their respective influences on the model’s performance in the context of our specific task.

The dice coefficient is a widely adopted metric in computer vision for assessing the similarity between two images. In our study, we utilize a modified version of the dice coefficient known as the dice loss, which served as a loss function for our model.

(1) $$\mathrm{DL}\left(y,\;p\right)=1-\frac{2\mathrm{yp}}{y+p}$$

where y is true value and p is the predicted outcome.

The focal Tversky loss is particularly well-suited for solving highly imbalanced class scenarios. It incorporates a γ coefficient that allows for the down-weighting of easy samples. Additionally, by adjusting the α and β coefficients, different weights can be assigned to false positives (FP) and false negatives (FN).

(2) $$\mathrm{FTL}\left(y,\;p\right)=(1-\frac{\mathrm{yp}}{\mathrm{yp}+\alpha \left(1-y\right)p+\beta y\left(1-p\right)}{)}^{\gamma }$$

where γ = 0.75, α = 0.3, and β = 0.7.

When performing the three-class segmentation task for the BG, NM, and M regions, calculating loss and back-propagating for the BG class is unnecessary and would make model training difficult. Therefore, during the execution of the three-class segmentation task, we do not calculate loss or perform backpropagation for the BG class.

### 3.8. Experimental Configuration and Evaluation Metrics

All experiments were conducted on four Intel Xeon Gold 6154 CPUs and a 32 GB Nvidia Tesla V100 GPU. The memory capacity configured was 90 GB. Our segmentation system was implemented in Python using Keras with TensorFlow 2.4.1.

The evaluation metrics employed in this study include precision (Equation (3)), sensitivity (Equation (4)), and the overall model assessment based on the F1-score (Equation (5)). The terms true positive (TP), false positive (FP), true negative (TN), and false negative (FN) were defined at the pixel level.

(3) $$\mathrm{Precision}=\frac{\mathrm{TP}}{\mathrm{TP}+\mathrm{FP}}$$

(4) $$\mathrm{Sensitivity}=\frac{\mathrm{TP}}{\mathrm{TP}+\mathrm{FN}}$$

(5) $$F1\text{-score}=2\times \frac{\mathrm{Precision}\times \mathrm{Sensitivity}}{\mathrm{Precision}+\mathrm{Sensitivity}}$$

## 4. Results

All experimental results in tables are obtained through 10-fold cross-validation, with a ratio of 8:1:1 for the training, validation, and testing sets, respectively. The learning rate used for training was 0.0001, batch size was set to 4, and the number of iterations was 500.

### 4.1. Negative Samples

**Figure 5:**
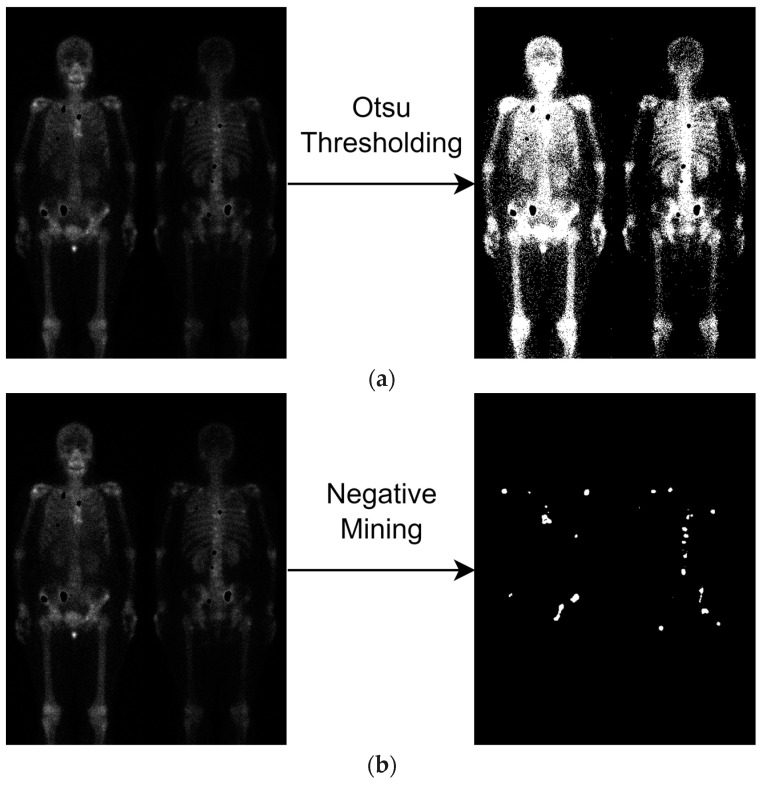
Illustration of negative sample productions. Notably, the metastasis hotspots are eliminated (the black holes), if the image has metastasis. (**a**) Otsu thresholding; (**b**) negative mining.

The qualitative results of two negative samples are illustrated in Figure 5. The Otsu thresholding can extract NM masks easily and produce three classes: BG, NM, and M. Its results are shown in Figure 5a. Nevertheless, negative mining requires two steps, as described in the method. Its results are shown in Figure 5b.

### 4.2. Results of the Baseline Network

The original Double U-Net network was trained using the D1 dataset and utilized the dice loss function. The objective of this experiment was to establish the baseline performance of the baseline network.

**Figure 6:**
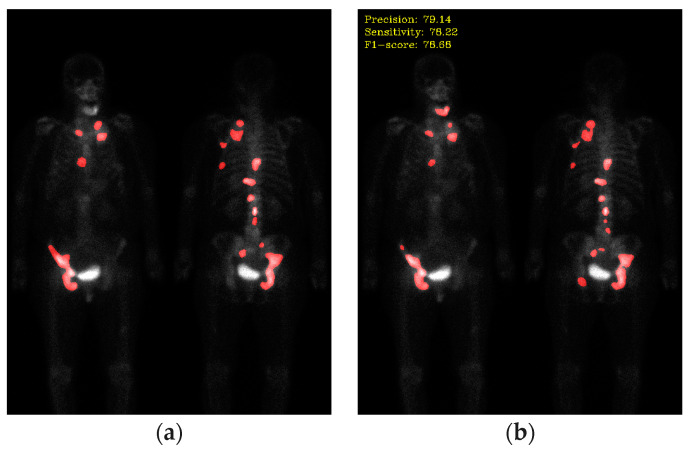
The qualitative result of the baseline network. (**a**) Ground truth; (**b**) segmentation results (precision: 79.14; sensitivity: 78.22; F1-score: 78.68).

For comparison, it is essential to evaluate the performance of deep learning models in each task using quantitative metrics. Here, the precision, sensitivity, and F1-score are utilized for performance evaluation. Figure 6 shows the qualitative results, and the quantitative results are shown in Table 2.

### 4.3. The Baseline Network Using Otsu Thresholding

**Figure 7:**
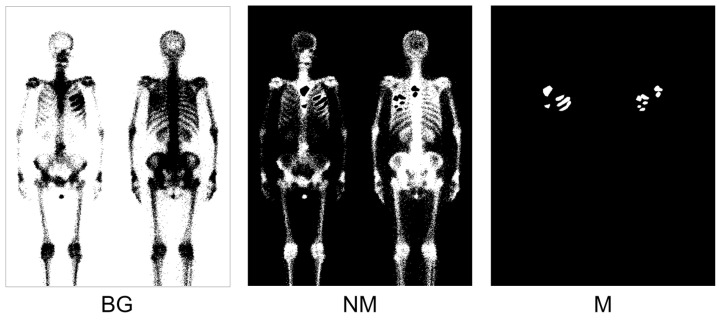
Illustration of applying Otsu thresholding to positive samples to generate NM masks. Three classes are included: BG, NM, and M.

The modified Double U-Net network was trained using the D1 dataset. Before training, we used the Otsu thresholding method on the D1 dataset for background pre-processing, generating the NM mask. Figure 7 illustrates a training sample from the D1 dataset, which includes three classes.

The model’s performance is shown in Table 3. In Table 3, we included the focal Tversky loss for comparison. Compared to Table 2, we observe that using the Otsu thresholding method for background pre-processing on the D1 dataset significantly improves the model’s performance. In both the dice loss and focal Tversky loss models, the F1-score improved by 3.12% and 4.16%, respectively.

**Figure 8:**
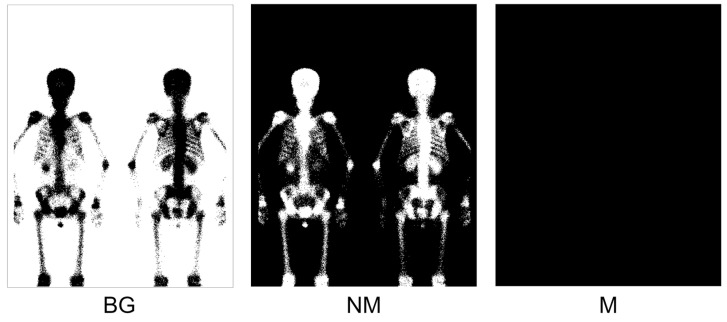
Illustration of applying Otsu thresholding to negative samples to generate NM masks. Three classes are included: BG, NM, and M.

We wanted to investigate the impact of adding negative samples to the training dataset on the model. In this experiment, we first used the Otsu thresholding method for background pre-processing on the D2 dataset, generating the NM mask. Then, we added the D2 dataset to the D1 training dataset in each fold. Figure 8 shows an example training sample from the D2 dataset, which contains three classes.

Table 4 presents the model performance when adding the D2 dataset using the Otsu thresholding method. In both the dice loss and focal Tversky loss models, precision improved by 2.61% and 2.09%, respectively. From the results, we did not observe any significant improvement in the F1 score. However, adding negative samples did indeed increase precision, which aligns with our expectations.

### 4.4. The Baseline Network Using Negative Mining

**Figure 9:**
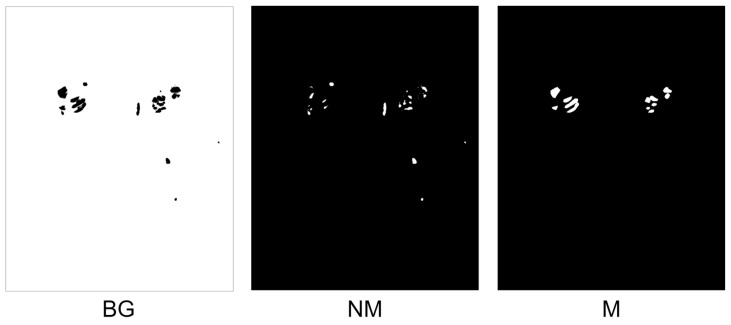
Illustration of applying negative mining to positive samples to generate NM masks. Three classes are included: BG, NM, and M.

The modified Double U-Net network was trained using the D1 dataset. Prior to training, we applied the negative mining method to pre-process the D1 dataset and generate the NM mask. Figure 9 illustrates a training sample from the D1 dataset, including three classes.

The model performance is shown in Table 5. Compared to the baseline (Table 2), negative mining indeed shows significant improvement. In the dice loss and focal Tversky loss models, F1-score improved by 2.14% and 2.27%, respectively.

**Figure 10:**
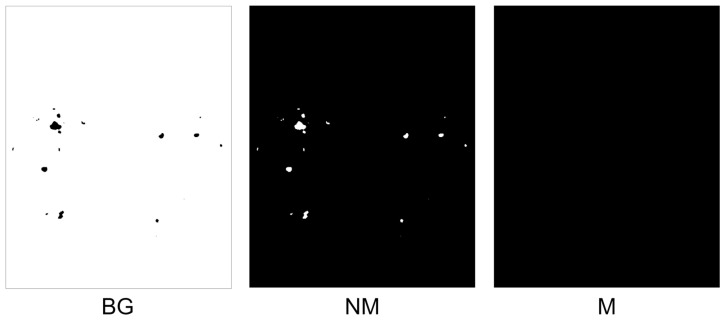
Illustration of applying negative mining to negative samples to generate NM masks. Three classes are included: BG, NM, and M.

Next, in this experiment, we first used the negative mining method to pre-process the D2 dataset and generate the NM mask. Then, we added the D2 dataset to the training data of D1 in each fold. Figure 10 shows a training sample from the D2 dataset (containing three classes).

The quantitative results of this experiment are shown in Table 6. In both the dice loss and focal Tversky loss models, precision improved by 1.85% and 1.64%, respectively. Similar to Table 4, adding negative samples to the training set led to a slight improvement in precision but a slight decrease in sensitivity. The F1-score remained unchanged, as expected.

### 4.5. Model Performance after Transfer Learning

Based on the previous experiments, we found that using the Otsu threshold method to generate the NM mask leads to better performance improvement. To understand the impact of transfer learning, we pre-trained the modified Double U-Net network using the D3 and D4 datasets. Before pretraining, we used the Otsu threshold method to pre-process the D3 and D4 datasets and generate the NM masks. Then, we added the D4 dataset to the training data of D3 in each fold.

The pre-trained model was fine-tuned by learning from breast cancer patient images. Before fine-tuning, we used the Otsu threshold method to pre-process the D1 and D2 datasets and generate the NM masks. Then, we added the D2 dataset to the training data of D1 in each fold.

**Figure 11:**
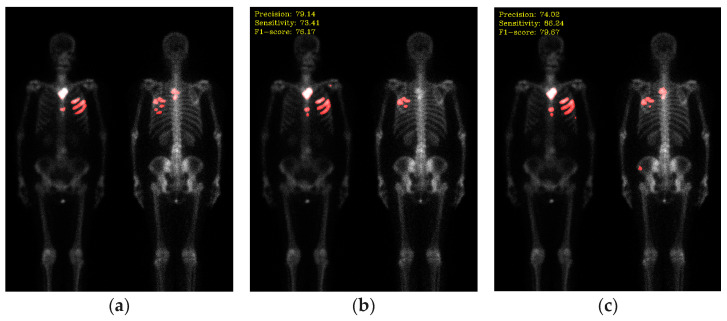
The qualitative results after transfer learning. (**a**) Ground truth; (**b**) segmentation results with dice loss (precision: 79.14, sensitivity: 73.41, F1-score: 76.17); (**c**) segmentation results with focal Tversky loss (precision: 74.02, sensitivity: 86.24, F1-score: 79.67).

The qualitative results of segmentation are shown in Figure 11. Specifically, we compare two loss functions: dice and focal Tversky.

The quantitative results are shown in Table 7. Compared to the results without transfer learning (Table 4), a slight improvement can be seen in the F1-score.

## 5. Discussion

In this study, the raw Double U-Net architecture served as the baseline model for performance comparison. Subsequently, two schemes on negative sample extraction are explored to see the impact on the model performance.

Otsu thresholding can easily separate air and body, thus removing the air background. The air background contains no information and wastes computation time. Although we define three classes in training, the BG class does not count into the loss. Another profit is to extract negative samples. In our previous study [14] we found that training with only metastasis (positive) class is not a good idea. It is better to train models with positive and negative samples simultaneously. Our results shown in Table 3 and Table 4 have confirmed this again; they are better than the baseline shown in Table 2.

Table 5 and Table 6 show the results obtained using negative mining. The model performances seem slightly worse than Otsu thresholding. This might be due to the fact that negative sample areas are significantly smaller than those negative samples produced using the thresholding technique. Thus, they contained less information for training. Moreover, negative mining requires a pre-trained model, and the thresholding technique does not. Our study indicates that while adding negative samples is necessary, there is not only one way to do so. There could be many other ways to create negative samples for training.

We compared our research with other relevant studies in terms of network architecture and results, as shown in Table 8. This table summarizes studies that used deep learning methods for the segmentation of bone metastases in bone scan images. The authors of [20] proposed an improved ResU-Net model for the segmentation of metastatic hotspots in thorax SPECT images, achieving precision, sensitivity, and IoU scores of 77.21%, 67.88%, and 61.03%, respectively. The authors of [21] added a methods attention mechanism to the original U-Net network’s skip connections to enhance model performance, resulting in an F1-score of 57.10% and an IoU of 63.30%. The authors of [16] introduced a neural network model based on U-Net++, achieving segmentation performance with a precision of 68.85%, a sensitivity of 62.57%, and an F1-score of 65.56%. The authors of [22] combined the UNet3+ network with an attention mechanism, proposing an improved UNet3+ network that achieved segmentation performance with a precision of 61.20%, a sensitivity of 68.33%, and an F1-score of 64.33%. The authors of [23] utilized a swin transformer as the backbone network and proposed a bone imaging focus segmentation algorithm, achieving an F1-score of 77.81% and an IoU of 35.59%.

In Table 8, the improved ResU-Net model used in [20] achieved the best model performance in the segmentation of metastatic lesions in the thorax region. The model based on the swin transformer in [23] achieved the highest F1-score. Our segmented region is the widest in Table 8, and our model’s F1-score of 66.60 is second only to [23].

The selection of loss function might play a crucial role in model performance. For complex tasks like segmentation, there is no universally applicable loss function. It largely depends on the properties of the training dataset, such as distribution, skewness, boundaries, etc. For segmentation tasks with extreme class imbalance, focal-related loss functions are more appropriate [41]. Additionally, since the vanilla Double U-Net model has a higher precision than sensitivity, we are keen to use Tversky-related loss functions to balance the false positives (FP) and false negatives (FN) rates. Therefore, we adopt focal Tversky loss as the compared loss function. In the future, further exploration and research should be conducted on the selection of optimizers.

**Figure 12:**
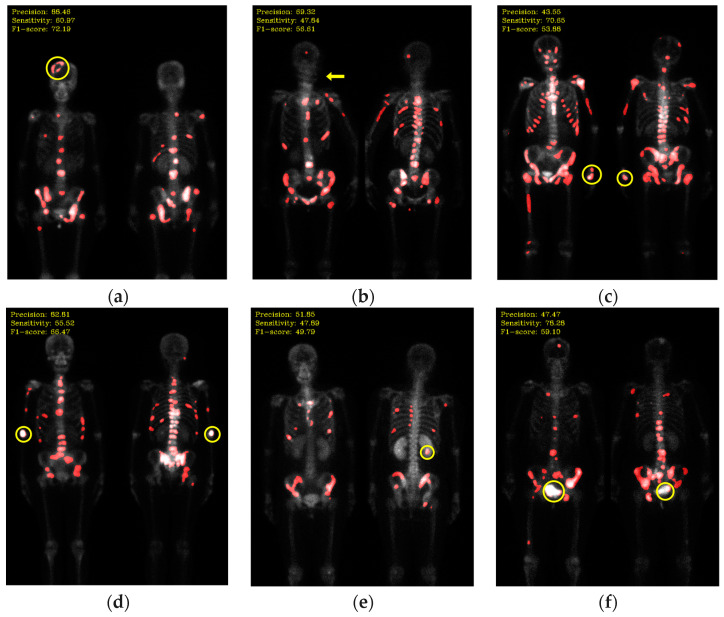
Mis-segmentation of non-metastatic lesions. (**a**) Bone fracture (head region) (precision: 88.46; sensitivity: 60.97; F1-score: 72.19); (**b**) motion artifact (head region) (precision: 69.32; sensitivity: 47.84; F1-score: 56.61); (**c**) injection site (wrist) (precision: 43.55; sensitivity: 70.65; F1-score: 53.88); (**d**) injection site (elbow) (precision: 82.81; sensitivity: 55.52; F1-score: 66.47); (**e**) kidney (precision: 51.85; sensitivity: 47.89; F1-score: 49.79); (**f**) bladder (precision: 47.47; sensitivity: 78.28; F1-score: 59.10).

**Figure 13:**
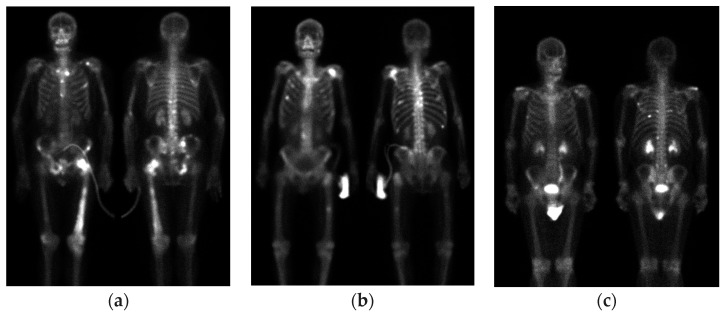
Artifacts in bone scan images of prostate cancer. (**a**) Catheter; (**b**) urinary bag; (**c**) diaper.

Not all hotspots in bone scan images represent bone metastases; normal bone absorption, renal metabolism, inflammation, and injuries can also cause hotspots in images, leading to false positives in segmentation. In addition to the inherent imaging principles of bone scan images that make training the model challenging, the presence of artifacts in the images is also a crucial factor leading to misclassification. Examples of such artifacts include high-activity areas like the kidneys and bladder, the injection site of the radioactive isotope, and motion artifacts, as shown in Figure 12. Apart from artifacts in breast cancer images, prostate cancer bone scan images also exhibit high-activity artifacts from catheters, urine bags, and diapers, as shown in Figure 13. In the future, appropriate pre-processing can be applied to minimize the impact from artifacts, or additional classes such as benign lesions and artifacts can be introduced to train the model more accurately.

The image pre-processing is usually important before using neural networks. Our previous study proposed a pre-processing method where the original images were combined into a 3D image to alleviate the issue of spatial connectivity loss [14]. View aggregation, an operation applied to bone scan images, has been used to enhance areas of high absorption [16]. This method enhances lesions that appear in both anterior and posterior view images and maps lesions that only appear in either anterior or posterior view images. However, that method cannot be applied in this study, since we calculate every pixel here and all errors (sensitivity, precision) are calculated in pixel-wise scale.

## 6. Conclusions

In this study, we confirm the validity of using negative samples in the task of bone metastatic lesions detection in breast cancer whole body bone scan images. The model is trained using positive and negative samples. We used background pre-processing to remove excess air background information. Adding negative samples improved the model’s precision. The images we used only excluded the less common regions below the knees for bone metastatic lesions and could simultaneously perform image segmentation for both anterior and posterior views. Our model is able to provide a more comprehensive evaluation of bone metastasis images. The precision, sensitivity, and F1-score for the segmentation of bone metastatic lesions are calculated on a pixel-level scale and the best results reach 70.24%, 61.80%, and 65.75% for dice loss and 69.96%, 63.55%, and 66.60% for focal Tversky loss, respectively.

The limitation of this study is the use of a small, single-center dataset, comprising only 100 breast cancer patients. This may result in limited model performance and generalizability. In the dataset, only 10 negative samples were collected from breast cancer patients, and the class imbalance between positive and negative samples could also pose a challenge to model performance.

There is still significant room for improvement in the model’s performance in this study. In the future, we plan to collect more WBBS images from different centers to further validate the proposed model’s performance. We will focus on fine-tuning the hyperparameters of the neural network and optimizing the choice of optimizers to enhance segmentation performance and reduce computational costs. Noise and artifacts in WBBS images are inevitable issues, and we plan to explore more image pre-processing methods to remove false artifacts and image noise to improve image quality, thus enhancing segmentation capabilities. Finally, we will use the interpretations of nuclear medicine physicians as the gold standard to compare the final model with the decisions made by nuclear medicine physicians, aiming to assess any discrepancies in decisions and evaluate the clinical utility of the model.

## Figures and Tables

**Table 1:** The details of the bone scan images.

	Breast Cancer	Prostate Cancer
w/metastasis	D1:90	D3:50
w/o metastasis	D2:10	D4:50
Total	100	100

**Table 2:** The quantitative results of the baseline network (dice loss).

Fold Number	Precision	Sensitivity	F1-Score
1	49.21	79.19	60.70
2	58.74	64.98	61.70
3	70.56	60.01	64.86
4	81.69	52.20	63.70
5	60.57	54.06	57.13
6	72.55	45.92	56.24
7	43.63	83.32	57.27
8	49.03	68.21	57.05
9	61.73	60.84	61.28
10	67.89	61.13	64.34
Mean	61.56	62.99	62.27

**Table 3:** The quantitative results for this experiment. Using the Otsu thresholding method for background pre-processing on the D1 dataset.

Fold Number	Dice Loss			Focal Tversky Loss		
	Precision	Sensitivity	F1-Score	Precision	Sensitivity	F1-Score
1	68.74	67.72	68.23	66.95	68.81	67.86
2	71.29	56.75	63.20	69.91	60.70	64.98
3	70.11	64.57	67.23	69.69	68.49	69.09
4	83.92	58.39	68.86	85.19	56.34	67.82
5	64.06	58.32	61.06	65.52	60.17	62.73
6	77.01	53.10	62.86	79.24	51.88	62.70
7	60.52	73.99	66.58	60.64	76.05	67.47
8	51.30	65.19	57.41	52.36	68.90	59.50
9	63.24	64.24	63.73	62.55	66.40	64.42
10	66.14	70.58	68.29	66.66	72.67	69.54
Mean	67.63	63.29	65.39	67.87	65.04	66.43

**Table 4:** The quantitative results for this experiment. Using the Otsu thresholding method for background pre-processing on the D2 dataset and adding it to the training set.

Fold Number	Dice Loss			Focal Tversky Loss		
	Precision	Sensitivity	F1-Score	Precision	Sensitivity	F1-Score
1	70.41	65.42	67.82	69.66	67.94	68.79
2	70.85	60.72	65.39	70.09	63.16	66.44
3	73.88	64.55	68.90	72.29	67.43	69.78
4	85.21	54.91	66.78	85.29	56.55	68.01
5	71.95	55.89	62.91	72.86	53.82	61.91
6	82.09	49.93	62.10	82.54	51.38	63.34
7	62.14	67.76	64.83	62.22	72.58	67.00
8	54.31	65.79	59.50	54.42	64.25	58.93
9	65.37	63.09	64.21	63.23	64.94	64.07
10	66.14	69.90	67.97	66.98	73.49	70.08
Mean	70.24	61.80	65.75	69.96	63.55	66.60

**Table 5:** The quantitative results for this experiment. Using the negative mining method for background pre-processing on the D1 dataset.

Fold Number	Dice Loss			Focal Tversky Loss		
	Precision	Sensitivity	F1-Score	Precision	Sensitivity	F1-Score
1	65.94	72.63	69.13	65.01	70.95	67.85
2	71.47	59.26	64.80	65.70	62.18	63.89
3	70.63	61.13	65.54	67.49	68.33	67.91
4	82.85	48.77	61.40	80.04	60.92	69.19
5	57.72	57.81	57.76	40.43	78.88	53.46
6	78.32	45.99	57.95	64.76	60.65	62.64
7	49.50	82.30	61.82	51.50	80.99	62.97
8	50.25	73.06	59.55	48.97	70.11	57.66
9	59.42	68.06	63.45	52.04	74.00	61.11
10	70.00	63.57	66.63	57.81	79.95	67.10
Mean	65.61	63.26	64.41	59.38	70.70	64.54

**Table 6:** The quantitative results for this experiment. Using the negative mining method for background pre-processing on the D2 dataset and adding it to the training set.

Fold Number	Dice Loss			Focal Tversky Loss		
	Precision	Sensitivity	F1-Score	Precision	Sensitivity	F1-Score
1	64.79	69.79	67.19	59.59	76.92	67.16
2	70.67	59.40	64.55	59.27	71.75	64.92
3	77.27	48.05	59.26	67.96	62.61	65.18
4	85.56	53.52	65.85	83.36	54.10	65.62
5	57.46	63.47	60.32	46.52	69.61	55.77
6	80.69	46.10	58.68	74.92	59.45	66.29
7	57.06	73.52	64.25	48.79	81.93	61.16
8	54.72	62.98	58.56	50.79	71.00	59.22
9	65.60	62.07	63.79	60.26	65.43	62.74
10	60.75	74.89	67.08	58.71	73.35	65.22
Mean	67.46	61.38	64.28	61.02	68.62	64.59

**Table 7:** The model performance with transfer learning.

Fold Number	Dice Loss			Focal Tversky Loss		
	Precision	Sensitivity	F1-Score	Precision	Sensitivity	F1-Score
1	67.81	70.28	69.02	66.51	72.04	69.17
2	71.44	63.75	67.38	70.80	60.64	65.33
3	76.75	60.73	67.80	69.87	69.25	69.56
4	86.56	51.54	64.61	81.80	60.28	69.41
5	68.88	62.84	65.72	51.28	66.78	58.01
6	84.14	43.46	57.31	80.00	52.20	63.18
7	62.07	66.34	64.14	50.69	86.04	63.80
8	51.90	72.17	60.38	43.82	85.62	57.97
9	62.30	64.44	63.35	52.07	77.78	62.38
10	64.98	74.47	69.40	63.92	77.56	70.09
Mean	69.68	63.00	66.17	63.08	70.82	66.72

**Table 8:** Comparison with network architecture and analysis results from related studies.

Method	Region	Precision	Sensitivity	F1-Score	IoU
ResU-Net [20]	Thorax	77.21	67.88	-	61.03
U-Net [21]	Thorax	-	-	57.10	63.30
U-Net++ [16]	Thorax	68.85	62.57	65.56	-
UNet3+ [22]	Thorax	61.20	68.33	64.33	-
Swin Transformer [23]	Thorax + Pelvis	-	-	77.81	35.59
Ours	Whole body excluded below knees	69.96	63.55	66.60	-

## Data Availability

Not applicable.
